# High Expression of PDLIM2 Predicts a Poor Prognosis in Prostate Cancer and Is Correlated with Epithelial-Mesenchymal Transition and Immune Cell Infiltration

**DOI:** 10.1155/2022/2922832

**Published:** 2022-06-06

**Authors:** Songzhe Piao, Lan Zheng, Haihong Zheng, Mengya Zhou, Qin Feng, Suna Zhou, Mang Ke, Haihua Yang, Xuequan Wang

**Affiliations:** ^1^Department of Urology, Taizhou Hospital of Zhejiang Province Affiliated with Wenzhou Medical University, Linhai, China; ^2^Department of Urology, Yanbian University Hospital, Yanji, China; ^3^Department of Obstetrics and Gynecology, Taizhou Hospital of Zhejiang Province Affiliated with Wenzhou Medical University, Linhai, China; ^4^Department of Pathology, Taizhou Hospital of Zhejiang Province Affiliated with Wenzhou Medical University, Taizhou, China; ^5^Department of Radiotherapy, Xi'an No. 3 Hospital, The Affiliated Hospital of Northwest University, Xi'an, China; ^6^Laboratory of Cellular and Molecular Radiation Oncology, Department of Radiation Oncology, Radiation Oncology Institute of Enze Medical Health Academy, Taizhou Hospital of Zhejiang Province Affiliated with Wenzhou Medical University, Taizhou, China

## Abstract

**Purpose:**

To elucidate the clinical and prognostic role of PDZ and LIM domain protein (PDLIM) genes and the association to epithelial-mesenchymal transition (EMT) and immune cell infiltration in patients with prostate cancer (PRAD).

**Methods:**

The data of RNA-seq, DNA methylation, and clinical features of PRAD patients were collected from The Cancer Genome Atlas (TCGA) database to define the prognostic value of PDLIM gene expression and the association with EMT and immune cell infiltration. A tissue microarray including 134 radical prostatectomy specimens was served as validation by immunohistochemistry (IHC) staining analysis.

**Results:**

The mRNA levels of PDLIM1/2/3/4/6/7 were significantly downregulated, while PDLIM5 was upregulated in PRAD (*P* < 0.05). High expression of PDLIM2 mRNA suggests poor progression free interval in PRAD patients. DNA methylation of PDLIM2 was correlated with its mRNA expression level, and that the cg22973076 methylation site in PDLIM2 was associated with shorter PFI (*P* < 0.05) in PRAD. Single-sample gene-set enrichment and gene functional enrichment results showed that PDLIM2 was correlated with EMT and immune processes. Spearman's test showed a significant correlation with six reported EMT signatures and several EMT signature-related genes. Tumor microenvironment analysis revealed that the PDLIM2 mRNA expression was positively correlated with the immune score, stromal score, and various tumor infiltrating immune cells. Additionally, the results showed that patients in the high-PDLIM2 mRNA expression group may be more sensitive to immune checkpoint blockade therapy. Finally, IHC analysis further implicated the protein level of PDLIM2 was upregulated in PRAD and acts as a novel potential biomarker in predicting tumor progression.

**Conclusion:**

Our study suggests that PDLIM family genes might be significantly correlated with oncogenesis and the progression of PRAD. PDLIM2 correlated with EMT and immune cell infiltration by acting as an oncogene in PRAD, which may serve as a potential prognostic biomarker for PRAD patients.

## 1. Introduction

Prostate cancer (PRAD) is a frequently diagnosed cancer type and a leading cause of cancer mortality in males worldwide [1]. Radical prostatectomy and radiotherapy are potentially curative strategies for localized PRAD, while androgen deprivation therapy (ADT) is the conventional treatment for metastatic PRAD. Unfortunately, despite high temporary response rates, cancer relapse often occurs in most patients and leads to deterioration into castration-resistant prostate cancer (CRPC). Treatment of metastatic, particularly CRPC, remains challenging. Emerging studies [2, 3] have shown that epithelial-mesenchymal transition (EMT) causes CRPC metastasis and development and enhances immunosuppression in the tumor microenvironment (TME). Currently, immunotherapy has gained wide attention for cancer treatment. However, the immunosuppressive tumor microenvironment limits the efficacy of immunotherapy [4]. Recently, studies [5, 6] have linked EMT-associated genes to immune components. However, the relationship between EMT activity and the efficacy of immunotherapy remains controversial. Some literatures [3, 5] suggested that patients with tumors exhibiting higher EMT-related gene expression may benefit from immune-checkpoint blockade (ICB) immunotherapy, while others have linked the expression of EMT regulators with immunotherapy resistance [6]. Therefore, further exploration of EMT-related genes and cancer immunotherapy may provide prognostic makers and potential therapeutic targets for patients with PRAD.

Currently, the PDLIM family comprises seven members, PDLIM1−7 [7]. The PDZ domain mediates protein binding and protein–protein interactions, thereby exerting various functions such as in cell proliferation, migration, polarity, EMT, and recognition of immune cells [8]. Recently, several studies reported that PDLIMs were linked with PRAD. PDLIM2 expression was abundant in CRPC-like cell lines but was not detectable in androgen-sensitive cell lines [9–11]. Furthermore, in vitro and in vivo results demonstrated that knockdown of PDLIM2 significantly inhibited tumor cell growth and invasiveness in human CRPC-like cells [9]. In contrast, PDLIM4 functions as a tumor suppressor and is hypermethylated and suppressed in PRAD cell lines compared to normal prostate cells [12–14]. Although research in prostate cancer has already given clues to the certain pathogenicity of PDLIMs, to date, the clinicopathological and prognostic value of PDLIM expression levels in PRAD remains unknown. In the present study, we aimed to elucidate the role of PDLIMs in EMT and immune cell infiltration, which can lead to a deeper understanding of the role of PDLIMs in PRAD, and more in-depth research in the future.

## 2. Materials and Methods

### 2.1. Data Acquisition and Expression Analysis of PDLIMs in PRAD

All RNA HTseq data in FPKM (fragments per kilobase of exon per million reads mapped) format including 551 PRAD tissues and 56 paired noncancerous normal tissues were obtained through The Cancer Genome Atlas (TCGA) database (https://portal.Gdc.cancer.gov/). DNA methylation data based on the Illumina Human Methylation 450 Bead chip and the clinicopathological data of TCGA-PRAD were also acquired from TCGA-PRAD dataset.

### 2.2. Survival Analysis of PDLIMs in PRAD

TCGA-PRAD tumor cohort was split into high and low groups for each PDLIM based on the median mRNA expression levels. Survival outcomes including overall survival (OS) and progression free interval (PFI) were estimated using the Kaplan–Meier and log-rank tests. Univariate and multivariate Cox proportional hazard models were used to assess the influence of PDLIMs on the survival outcomes of PRAD patients. The PFI events in this study were based on data from TCGA database, as described by Liu et al. [15]. Patients without survival data were excluded from the survival analysis.

### 2.3. Mutation Data Analysis of PDLIMs in PRAD

To identify driver mutated genes in TCGA-PRAD cohort, we adopted the R package TCGA mutations [16]. Additionally, the tumor mutation burden (TMB) of each TCGA-PRAD sample was calculated using a previously reported method [17]. In this study, tumor mutation burden (TMB) for TCGA cohorts is obtained from TCGA MC3 study. For consistency, TMB is estimated by restricting variants within Agilent Sureselect capture kit of size 35.8 MB. A Wilcoxon test was applied to compare the TMB distribution differences between the high and low-PDLIM2 mRNA expression groups. Furthermore, a Wilcoxon test was used to compare the mRNA expression levels of PDLIM2 between mutated and wild-type (WT) cases, using the Tumor Immune Estimation Resource (TIMER) 2.0 [18] database (http://timer.cistrome.org/).

### 2.4. Prognostic Potential of DNA Methylation of PDLIM2 Loci in PRAD

DNA methylation samples were divided into low and high groups based on the median methylation beta-value of each locus in PDLIM2. The Kaplan–Meier method was used to assess the association between the methylation status of PDLIM2 and PFI. The correlation between each methylation level of the selected loci and the mRNA expression of PDLIM2 was determined with the “corrplot” R package.

### 2.5. Functional Enrichment Analysis of PDLIM2 Coexpression Networks

PDLIM2 coexpressed genes in TCGA-PRAD were identified using Spearman's rank correlation (∣*r* | >0.4, *P* < 0.05). Functional enrichment analysis was performed to predict the function of PDLIM2 coexpressed genes using the online Metascape tool [19].

### 2.6. Gene-Set Enrichment Analysis of Published EMT Signatures

Single-sample gene-set enrichment analysis (ssGSEA) methods [20] were used to score individual samples against the EMT status based on previously reported findings in PRAD or pancancer. Furthermore, the EMT signature score and the EMT signature-related genes that were highly related to PDLIM2 were visualized using the online Evenn website [21]. Spearman's correlation coefficients were employed to explore the correlation between PDLIM2 and different EMT signature enrichment scores and EMT signature-related genes. The EMT scores from different published EMT signatures and the normalized expression level of each EMT signature-related gene that was significantly related to PDLIM2 are shown in the heatmap constructed using the “pheatmap” package (v_1.0.8) (https://CRAN.R-project.org/package=pheatmap). Kaplan–Meier curves combined with a log-rank test for PFI were constructed to predict the value of the signature using the R package “survival.” The expression correlation between PDLIM2 and different EMT signature intersection genes was visualized using the package “ggstatsplot” (https://CRAN.R-project.org/package=ggstatsplot) in R.

### 2.7. Tumor Microenvironment Analysis

Immune infiltration analysis of PRAD was assessed using the xCell method from the TIMER 2.0 database (http://timer.cistrome.org/) [18]. To identify the correlation between PDLIM2 and the infiltration levels of various immune cells [22], Spearman and Wilcoxon rank sum tests were performed to explore *P* values. The immune checkpoint gene expression differences between the high and low of PDLIM2 expression groups were analyzed. The detailed relationships between PDLIM2 and hematopoietic stem cells, cancer associated fibroblasts, immune scores, and microenvironment scores were also plotted using the ggscatterstats function in the “ggstatsplot” package.

### 2.8. Correlation between PDLIM2 mRNA Expression and Biomarkers for Predicting Immunotherapy Response

The potential immunotherapy response prediction between PDLIM2 mRNA high and low groups was estimated with immune checkpoint genes and mismatch repair (MMR) genes. The expression levels of eight representative immune checkpoint genes, namely, SIGLEC15, TIGIT, CD274, HAVCR2, PDCD1, CTLA4, LAG3, and PDCD1LG2, were selected to explore differences in the mRNA expression of above eight genes between the PDLIM high and low groups via a Wilcoxon test. The association between PDLIM2 expression and MMR genes (MLH1, MSH2, MSH6, PMS2, and EPCAM) was evaluated using the Spearman method.

### 2.9. Immunohistochemical (IHC) Analysis

For IHC analysis, a tissue microarray (TMA) containing 134 PRAD tissue samples and 31 adjacent tissue samples was purchased from Shanghai OUTDO Biotech Co. (Shanghai, China; HproA180PG09). The correlation between PDLIM2 protein expression and clinicopathological parameters, such as age, PSA value, Gleason score, pathology TNM stage, and various biomarkers of PRAD patients was also analyzed. Immunostaining was performed using the EnVision Flex+ system (K8002, Dako, Glostrup, Denmark). Immunohistochemical analysis was performed as previously described [23]. Specimens were incubated with PDLIM2 primary antibody (1 : 200, Invirtrogen, MA5-25512) at 4°C overnight. The results were analyzed by two pathologists (ZHH and ZMY). The immunostaining for PDLIM2 was semiquantitatively scored according to the intensity of cell staining and the proportion of stained tumor cells. Only a cytoplasmic expression pattern was considered as positive staining. Briefly, the staining intensity was scored as “0” (negative, no staining), “1” (weak staining), “2” (moderate staining), or “3” (strong staining). If the total score (percentage score × intensity score) was 1 or less, PDLIM2 protein expression was considered low, and if the total score was more than 1, the samples were considered to have high-PDLIM2 expression.

### 2.10. Statistical Analysis

Statistical analysis was performed using R (version 4.03) and R studio (version 1.2.5042) software. Numerical data are described as the median and/or range; intergroup comparisons were performed with a Wilcoxon signed-rank test or Mann–Whitney *U* test. For categorical data, we used a chi-square test or Fisher exact test to perform intergroup comparisons. The Kaplan–Meier method and univariant and multivariant Cox regression tests were performed using the survival package. Correlation coefficients were determined via Spearman correlation analysis, and ∣*r* | >0.40 and *P* < 0.05 were considered significant. The relationship between PDLIM2 expression and clinicopathologic features was evaluated with a Wilcoxon rank sum test, chi-square test, or Fisher's exact test. *P* values less than 0.05 for a two-tailed test were accepted as statistically significant.

## 3. Results

### 3.1. Dysregulation of PDLIM Genes in PRAD

**Figure 1:**
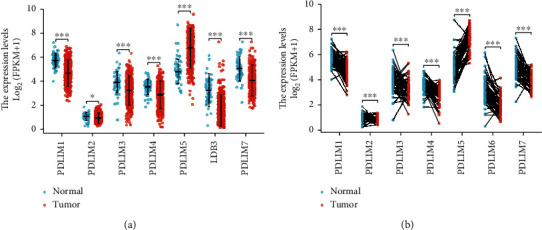
Expression patterns of PDLIMs in prostate cancer. (a) The expression of PDLIM family genes in prostate cancer tissues (*n* = 499) compared to normal adjacent tissues (*n* = 42) in in TCGA-PRAD cohort. (b) Expression of PDLIM family genes in paired prostate cancer tissues and normal tissues (52 vs. 52). ^∗^*P* < 0.05, ^∗∗^*P* < 0.01, and ^∗∗∗^*P* < 0.0001.

To investigate the mRNA expression levels of PDLIMs in PRAD, PDLIM mRNA expression between PRAD and normal samples was evaluated using unpaired (*n* = 551) and paired (*n* = 56) data from TCGA database. The results showed that PDLIM5 mRNA expression was upregulated in the PRAD group compared to the normal group, but PDLIM1/2/3/4/6/7 were downregulated in both unpaired (Figure 1(a)) and paired samples (Figure 1(b)).

### 3.2. High Expression of PDLIM2 mRNA in PRAD Is Correlated with Poor Prognosis

**Figure 2:**
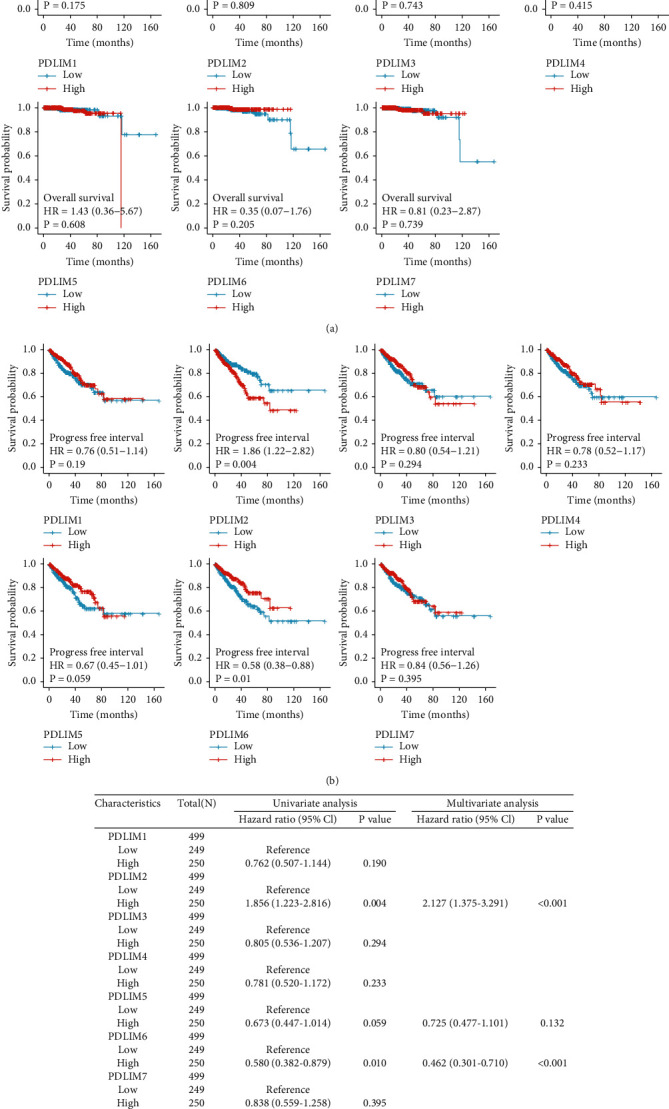
High-PDLIM2 expression was an independent predictor of poor prognosis in prostate cancer. (a, b) Kaplan–Meier survival plots of overall survival (OS) and progression free interval (PFI). (c) Univariate and multivariate Cox proportional analyses for PFI.

Kaplan–Meier plotter showed that high-PDLIM2 expression was correlated with poor PFI (*P* = 0.004), but high PDLIM6 expression was correlated with favorable PFI (*P* = 0.01) (Figure 2(b)). However, the other PDLIM genes showed no significant effect on OS or PFI in PRAD patients (Figures 2(a) and 2(b)). Univariate Cox analysis (HR = 1.856, 95% CI: 1.223-2.816, and *P* = 0.004) and multivariate Cox analysis (HR = 2.127, 95% CI: 1.375-4.291, and *P* < 0.001) indicated that PDLIM2 was a risk prognostic factor for PFI, while PDLIM6 (HR < 1, *P* < 0.05) was identified as a favorable prognostic factors in PRAD patients (Figure 2(c)). Thus, PDLIM2 was selected for further analysis in this study.

### 3.3. Correlations between PDLIM2 mRNA Expression and Clinicopathological Parameters of PRAD

A total of 499 primary PRAD patients with both clinical and gene expression data from TCGA-PRAD cohort were enrolled (Table 1). According to the median value of PDLIM2 mRNA expression, the patients were divided into high (*n* = 250) and low (*n* = 249) expression groups according to the median value of PDLIM2 mRNA expression. A chi-square test or Fisher's exact test showed that PDLIM2 mRNA expression was correlated with primary therapy outcome (*P* < 0.001) and Gleason score (*P* = 0.003).

### 3.4. Somatic Mutation Landscape

**Figure 3:**
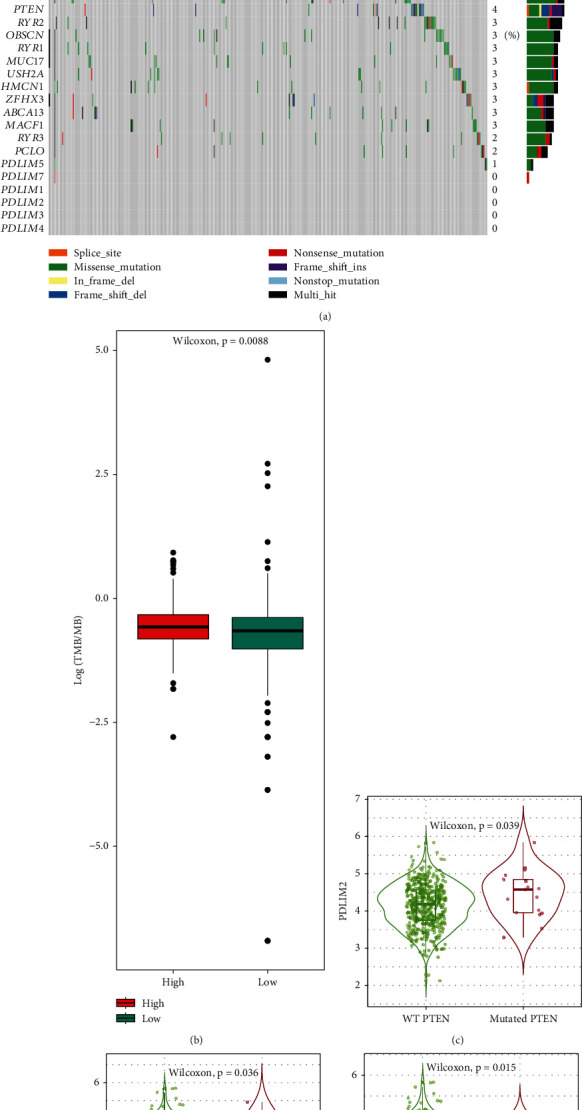
Correlation between PDLIM2 mRNA expression and mutation frequencies of prostate cancer-associated genes. (a) Gene mutation frequencies in TCGA-PRAD. (b) TMB scores of the high- and low-PDLIM2 groups using data from TCGA database. (c–e) Violin plots examining the effect of PTEN, FOXA1, and TTN gene mutations on PDLIM2 mRNA expression in prostate cancer.

TCGA-PRAD somatic mutation data showed that TP53, SPOP, TTN, MUC16, KMT2D, and FOXA1 were the top six genes with the highest genetic mutations, and the mutation rates were 12, 11, 11, 6, and 6%, respectively (Figure 3(a)). TMB, immune checkpoint genes, and impaired DNA MMR have been found to affect the response of cancer cells to ICB treatment [24]. The PDLIM2 high expression group showed lower TMB than the low expression group (*P* < 0.01, *n* = 498) (Figure 3(b)). To determine the correlations between these gene mutation phenotypes and PDLIM2 expression, the TIMER 2.0 database [18] was applied using a Wilcoxon rank sum test. In this cohort, patients with PTEN-mutated tumors had higher PDLIM2 mRNA expression levels than patients with PTEN wild-type (WT) tumors (*P* = 0.039). In contrast, increased PDLIM2 mRNA expression was observed in patients with WT-FOXA1 (*P* = 0.036) and WT-TTN (*P* = 0.015) compared with those with mutations (Figures 3(c)–3(e)), indicating that mutation of PTEN, FOXA1, and TTN showed a significant influence on aberrant PDLIM2 expression. No significant differential PDLIM2 expression could be found between the WT and mutant genes for SPOP, KMT2D, or MUC16 (*P* > 0.05) (data not shown).

### 3.5. DNA Methylation of PDLIM2 in PRAD

**Figure 4:**
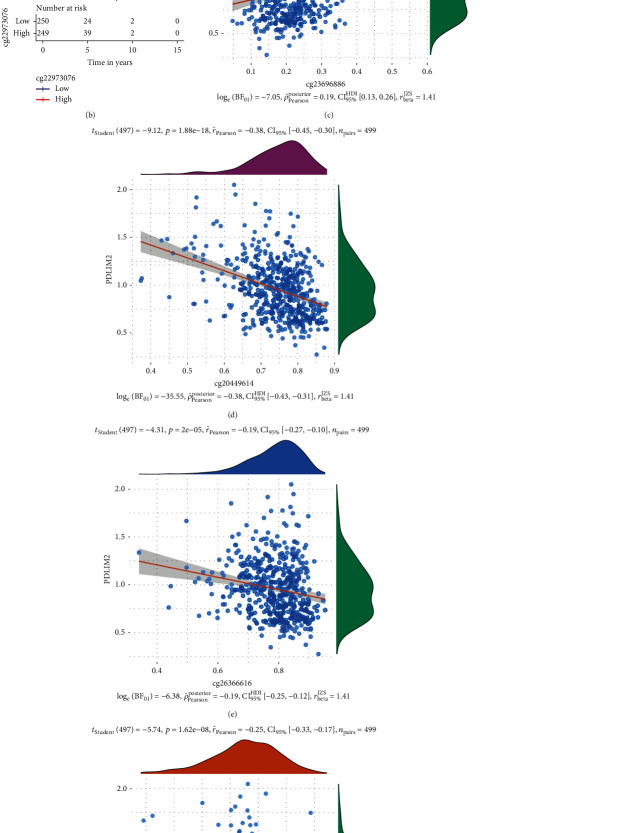
DNA methylation of PDLIM2 in TCGA-PRAD cohort. (a) The correlation between PDLIM2 methylation sites and PDLIM2 expression in patients with prostate cancer. Blue represents a negative correlation; red represents a positive correlation; “×” represents no statistical significance. (b) Kaplan–Meier survival analysis showed that the cg22973076 methylation site in PDLIM2 was correlated with poor PFI in prostate cancer patients (*P* < 0.05). (c–g) Spearman's correlation analysis showed a correlation between the PDLIM2 mRNA expression level and PDLIM2 methylation sites in prostate cancer patients.

As previous studies reported that PDLIM mRNA expression levels are regulated by DNA methylation in many cancer types [12, 25], and we further investigated the DNA methylation on PDLIM2 mRNA expression and survival in PRAD patients. The PDLIM2 mRNA expression was positively correlated with the methylation sites cg23696886 and negatively correlated with methylation site of cg20449614, cg26366616, cg05698069, and cg22973076 (Figures 4(a) and 4(c)–4(g)). The cg22973076 methylation site in PDLIM2 was correlated with poor PFI in PRAD patients (Figure 4(b)).

### 3.6. Identification of PDLIM2 Coexpressed Genes and Their Functional Enrichment

**Figure 5:**
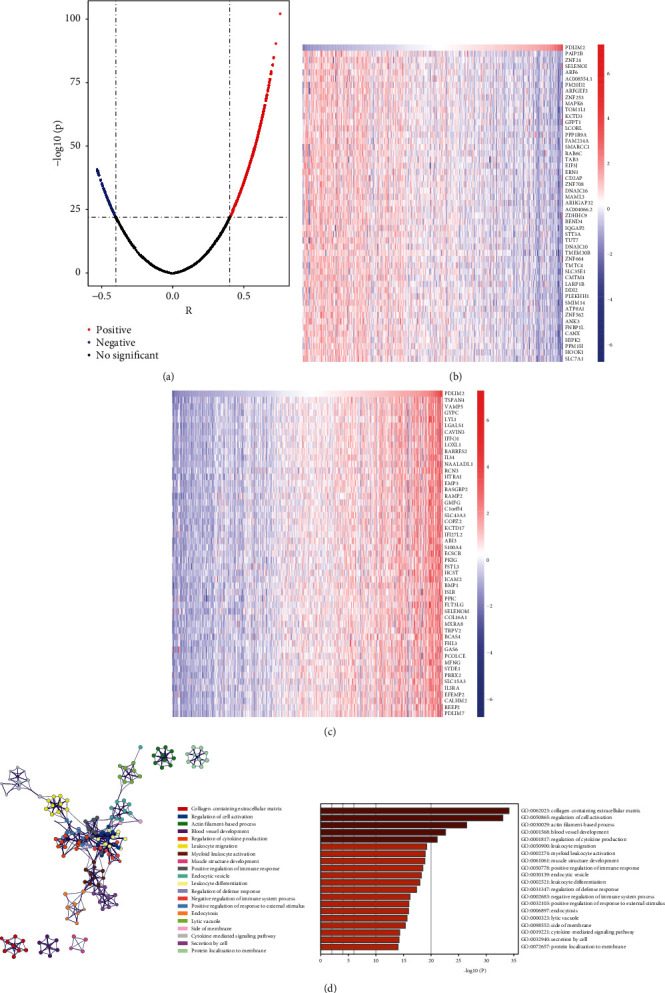
Enrichment analysis of the coexpression genes of PDLIM2. (a) Spearman's correlation analysis showed a correlation between PDLIM2 and differentially expressed genes in prostate cancer. (b, c) Heatmap showing the top 50 significant genes negatively and positively correlated with PDLIM2 in TCGA-PRAD. (d) Enrichment analysis revealed the biological processes involved in the PDLIM2-related coexpressed genes.

The genes positively (*r* > 0.4, *P* < 0.05, and *n* = 1352) and negatively (*r* < 0.4, *P* < 0.05, and *n* = 225) correlated with PDLIM2 were selected for enrichment analysis. As shown in Figure 5(a), the red dots and blue dots indicate upregulated and downregulated genes, respectively. The expression of the top 50 genes that were most positively (including PAIP2B, ZNF24, and SELENOI) and negatively (including TSPAN4, VAMP5, and GYPC) associated with PDLIM2 is shown in the heatmap (Figures 5(b) and 5(c)). Furthermore, gene functional enrichment analysis showed that PDLIM2 coexpressed genes were correlated with the regulation of cell activation, collagen-containing extracellular matrix, regulation of cytokine production, actin filament-based process, and blood vessel development among other processes (Figure 5(d)). It is important to note that PDLIM2 is also involved in the positive regulation of immune response or cell migration and in negative regulation of the immune system process and leukocyte differentiation.

### 3.7. Correlation Analysis between PDLIM2 Expression and the EMT Process

**Figure 6:**
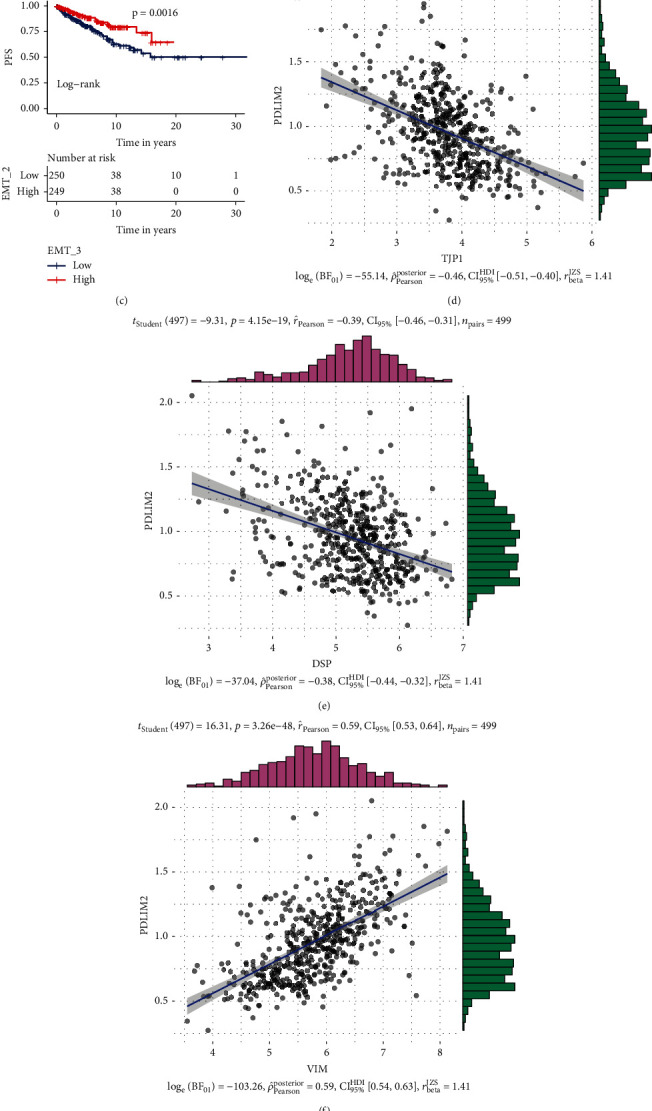
PDLIM2 mRNA is involved in EMT process in prostate cancer patients. (a) The mRNA expression level of PDLIM2 was correlated with various EMT signatures and EMT-related genes. EMT_1, Chen et al. 2013; EMT_2, Klarmann et al. 2009; EMT_3, Mathews et al. 2010; EMT_4, Schell et al. 2016; EMT_5, Sethi et al. 2010; EMT_6, Stylilanou et al. 2019. (b) Heatmap showing the relationship between EMT signature-related genes and PDLIM2 expression. (c) Kaplan–Meier survival analysis showed the EMT signature reported by Mathews et al. was significantly associated with poor PFI in patients with prostate cancer. (d–i) The correlation between PDLIM2 mRNA expression land EMT biomarkers, including TJP1, DSP, VIM, CDH1, OCLN, and MMP2 in prostate cancer patients.

Using six previously published EMT signatures [26–31], we found that PDLIM2 was significantly correlated with EMT and the EMT signature-related genes (Figure 6(a)). Furthermore, the relationship between EMT signature-related genes and PDLIM2 expression is shown directly in a heatmap (Figure 6(b)). Additionally, the EMT signature reported by Mathews et al. [28] was significantly associated with poor PFI in PRAD (Figure 6(c)). In addition, PDLIM2 showed positive correlation with the EMT markers of vimentin (VIM) and matrix metallopeptidases (MMP2) and a negative correlation with tight junction protein 1 (TJP1), desmoplakin (DSP), E-cadherin (CDH1), and occludin (OCLN) (Figures 6(d)–6(i)).

### 3.8. Correlation Analysis between PDLIM2 Expression and Immune Infiltration

**Figure 7:**
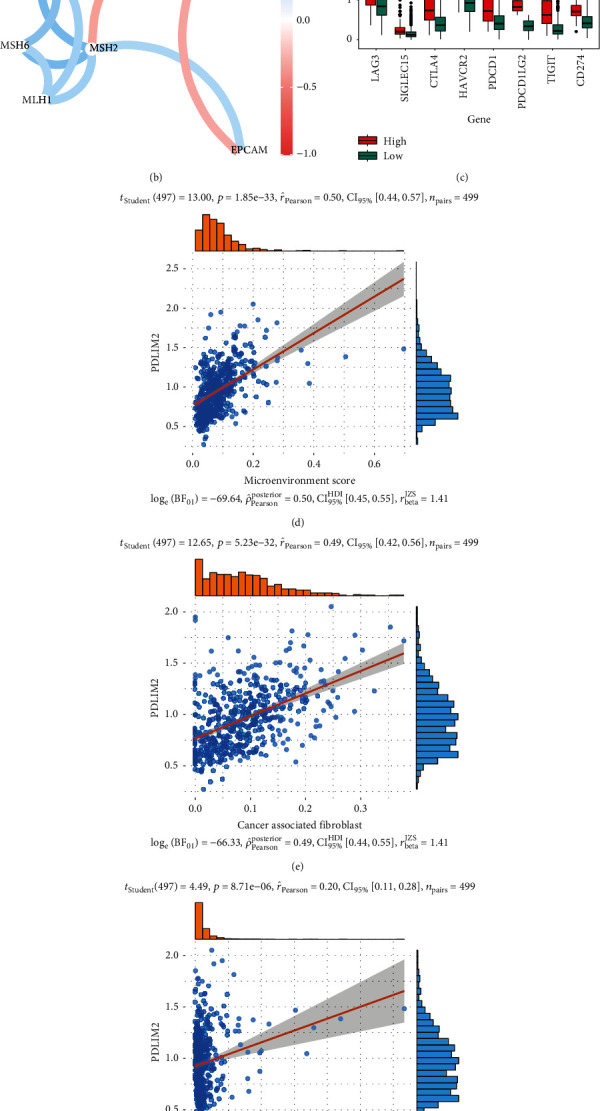
PDLIM2 mRNA was involved in immune infiltration in patients with prostate cancer. (a) The mRNA expression level of PDLIM2 was correlated with immune infiltration levels in prostate cancer patients. Blue represents a negative correlation; red represents a positive correlation; “×” represents no statistical significance. (b) The relationship between PDLIM2 mRNA expression and MMR genes. (c) Relationships between PDLIM2 mRNA expression and immune checkpoints. (d–h) Spearman's correlation analysis showed the correlation between PDLIM2 mRNA expression and microenvironment score, cancer-associated fibroblast, immune score, hematopoietic stem cell, and stroma score. ^∗^*P* < 0.05, ^∗∗^*P* < 0.01, and ^∗∗∗^*P* < 0.0001.

Immune cells in the tumor microenvironment play a vital role in the progression of PRAD [32, 33], and PDLIM2 has been reported [34] to participate in immune process. Therefore, whether PDLIM2 expression was correlated with immune infiltration levels in PRAD was investigated via Spearman's correlation analysis in the pancaner TCGA database through the “xCell” algorithm [35]. The results revealed that PDLIM2 expression was positively correlated with immune score, stromal score, microenvironment score, common myeloid progenitors (CMPs), effector memory CD4+ T cell, CD4+ type-1-helper (Th1) T cell, CD4+ (nonregulatory) T cell, naïve CD4+ T cell, CD8+ T cell, central memory CD8+ T cell, T cell NK, monocyte, myeloid dendritic cell (mDC), myeloid dendritic cell activated, macrophage M1, macrophage M2, B cell, naïve B cell, memory B cell, granulocyte-monocyte progenitor (GMP), endothelial cell, hematopoietic stem cell (HSC), and cancer-associated fibroblast (CAF), while PDLIM2 expression was negatively correlated with common lymphoid progenitor (CLP), central memory CD4+ T cell, memory CD4+ T cell, master cell, plasma B cell, and regulatory T cell (Treg) (Figures 7(a) and 7(d)–7(h)). These results provide evidence for the impact of PDLIM2 on immune infiltration in PRAD.

### 3.9. PDLIM2 Expression Could Predict the Immunotherapeutic Response of PRAD

The PDLIM2 high expression group showed higher mRNA expression levels of checkpoint genes, including SIGLEC15, TIGIT, CD274, HAVCR2, PDCD1, CTLA4, LAG3, and PDCD1LG2 (*P* < 0.05) (Figure 7(c)). Moreover, PDLIM2 mRNA expression was negatively correlated with MMR genes, including MSH2, EPCAM, and PMS2 (*r* < −0.3, *P* < 0.05) (Figure 7(b)), indicating that PDLIM2 may play a role in PRAD by regulating MMR genes. These results indicate that the high-PDLIM2 groups may be more sensitive to ICB therapy.

### 3.10. Immunohistochemistry Analysis of PDLIM2 in PRAD

**Figure 8:**
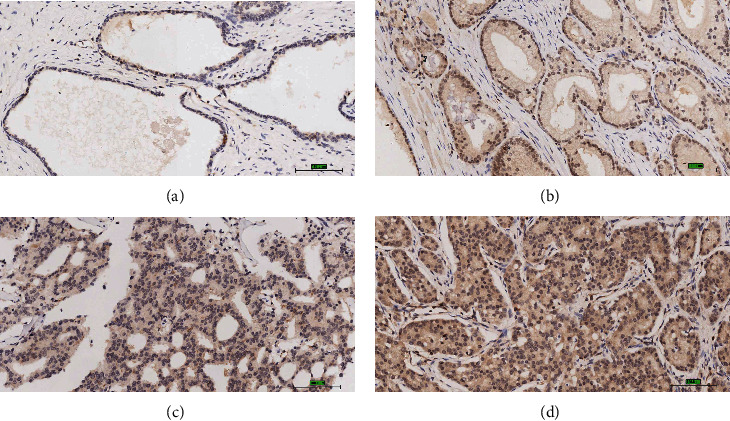
IHC staining of PDLIM2 in prostate cancer samples. (a) Protein expression of PDLIM2 in normal prostate tissues; (b) weak, (c) moderate, and (d) strong positive expression of PDLIM2 protein in prostate cancer tissues.

Next, we conducted immunohistochemical (IHC) assays in an independent cohort of PRAD tissue slices to determine the protein level of PDLIM2. Representative staining of PDLIM2 in PRAD tissue is shown in Figure 8. The PDLIM2 was mainly observed in the cytosol, and low expression levels were detected in the nucleus. As shown in Figure 8 and Table 2, the rate of positive PDLIM2 expression was significantly higher in PRAD tissues (32.3%, 10/31) than in tumor-adjacent tissues (9.7%, 3/28) (*P* = 0.029). The expression of PDLIM2 protein was significantly higher in Gleason 7 (57.14%, 24/42) or >7 (43.75%, 28/64) PRAD tissue than in tissues scored Gleason 6 (33.33%, 7/28). Furthermore, higher PDLIM2 levels were also correlated with advanced T status and advanced N status in PRAD patients. The expression levels of PDLIM2 protein were higher in T4 (64.71%, 11/17) than in T1-3 (36.74%, 18/49) cases (*P* = 0.045). For the N status, the high expression rate of PDLIM2 protein was 65.0% (13/20) in the N1 PRAD patients, but only 40.71% (46/113) in N0 cases (*P* = 0.044). However, high expression of PDLIM2 protein was not correlated with age or various tumor biomarkers in patients with PRAD (*P* > 0.05), except Ki67 (*P* = 0.034) (Table 3). These results illustrate that the patients with higher PDLIM2 expression suffer from a high risk of developing more advanced prostate tumors than those with lower PDLIM2 expression, suggesting a close association between this protein and the clinicopathological features of PRAD.

## 4. Discussion

Emerging evidence has demonstrated that PDLIMs are dysregulated in variety of tumors and are involved in various cellular and intercellular processes. For instance, PDLIM1 knockdown induces EMT and promotes invasion and metastasis in hepatocellular carcinoma (HCC) cells [36]; overexpression of PDLIM2 and PDLIM7 could serve as adverse prognostic factors for acute myeloid leukemia [37]; downregulation of PDLIM4 is correlated with aggressive tumor growth and poor prognosis in ovarian cancer patients [38]; PDLIM5 knockdown inhibited TGF*β*-signaling and TGF*β*-induced epithelial-mesenchymal transition [39]. However, to date, the association between PDLIM expression and clinicopathological features and prognosis of PRAD has not been elucidated. The present study found that PDLIM1, PDLIM2, PDLIM3, PDLIM4, PDLIM6, and PDLIM7 mRNA levels were decreased but that the PDLIM5 mRNA level was increased in both unpaired and paired samples in TCGA-PRAD cohort. Based on TCGA-PRAD data, among those PDLIMs, only PDLIM2 overexpression showed a significant correlation with adverse PFI. Moreover, the univariate and multivariate Cox proportional hazard regression models identified that PDLIM2 was an effective independent prognostic factor affecting PFI in patients with PRAD. These results suggest that PDLIM2 is a potential biomarker for predicting the prognosis of PRAD patients.

Through IHC staining of PDLIM2 protein, we found that the rate of positive PDLIM2 protein expression was higher in PRAD tissues than in matched normal tissues. The inconsistency between the mRNA and protein expression levels of PDLIM2 in PRAD tissues compared to normal tissues might be related to a variety of reasons. First, a recent study [40] reported that PDLIM2 mRNA downregulation is associated with promoter hypermethylation. In this study, we found that the methylation status of four methylation sites (cg20449614, cg26366616, cg05698069, and cg22973076) of PDLIM2 showed a negative correlation with the gene expression. Therefore, we infer that DNA methylation might also be an important epigenetic mechanism for PDLIM2 mRNA downregulation in PRAD. Additionally, the cg22973076 methylation site in PDLIM2 was correlated with poor PFI in PRAD patients. Remarkably, this association was found in a nonlinear relationship. This phenomenon may be due to the fact that methylation within a gene-body or transcribed region may not affect the expression of the whole gene [41, 42]. Moreover, there may be a combined regulatory role of different methylation sites exist. These findings suggest that epigenetic contributions to transcriptional regulation occur in a more complex and dynamic manner. Further studies are needed to prove that causal link. Additionally, the process may be regulated by many additional factors, such as posttranscription and translation levels, protein modification, protein–protein interactions, and other regulatory mechanisms [43]. Finally, there were more patients with high mRNA expression (normal = 52 and primary tumor = 499) than patients with protein expression (normal = 31 and primary tumor = 144). Thus, additional studies are warranted to confirm the present results. Such possibilities require further studies. The high-PDLIM2 mRNA expression group was more likely to be patients of with higher Gleason scores and primary treatment outcome. We found that high-PDLIM2 protein expression was correlated with higher Gleason scores, advanced T and N status, and higher Ki67 protein expression. Taken together, these results strongly suggest the PDLIM2 protein may play an oncogenic role in PRAD. These findings were consistent with previous results [9].

The function of PDLIM2 in tumors remains controversial. Paradoxically, PDLIM2 can be either a tumor suppressor or a tumor promoter, depending on the cellular context. A previous study [44] showed that upregulation of PDLIM2 in cancer cells impaired anchorage-independent growth both in vitro and in vivo, indicating a tumor-suppressive role. On the other hand, PDLIM2 is overexpressed in a variety of cancer types, including PRAD, in which it promotes cell proliferation, malignant transformation, and EMT, supporting its prooncogenic roles [9]. Therefore, PDIM2 may represent a potential therapeutic target in human malignancies. TP53, SPOP, TTN, MUC16, KMT2D, and FOXA1 were the top six most commonly mutated genes in TCGA-PRAD cohort in the present study. PDLIM2 mRNA expression was significantly correlated with PTEN, FOXA1, and TTN mutations. The PTEN gene is frequently mutationally inactivated in prostate cancer [45]. PTEN loss leads to enhanced cell proliferation and migration as well as castration-resistant growth [46]. Androgen receptor (AR) is the essential driver and therapeutic target in prostate cancer. FOXA1 mutants promote AR binding and significant AR transcriptional activity [47]. Additionally, FOXA1 mutants can also induce EMT and enhance cancer metastasis. In recent years, studies examining the relationship between TTN mutation and the immunotherapy response of solid tumors have been widely reported [48]. The relationship between PDLIM2 and the above top mutated genes in PRAD may help to elucidate the underlying molecular mechanisms. To confirm this inference, further investigation is needed.

Of note, enrichment analysis showed that PDLIM2 coexpressed genes were mainly related to the collagen-containing extracellular matrix and positive regulation of cell migration. Previous studies have found that PDLIM2 regulates EMT. PDLIM2 is expressed in epithelial cells, may be repressed in cancer, and is highly expressed in cancer cells that exhibit an EMT phenotype [49–54]. Suppression of PDLIM2 in invasive PRAD (DU145) and breast cancer cells (MDA-MB-231) causes increased E-cadherin expression and cell–cell contact, loss of directional migration, altered expression and activity of many transcription factors associated with tumorigenesis, and reversal of EMT [50]. Thus, we explored the relationship between PDLIM2 and the EMT-related gene signature in PRAD based on six published studies [26–31]. The results showed that PDLIM2 mRNA expression was correlated with EMT signature scores and EMT-related genes. PDLIM2 showed a positive correlation with mesenchymal markers (VIM and MMP2) and a negative correlation with epithelial markers (TJP1, DSP, CDH1, and OCLN) in EMT. This result is consistent with a previous report [9], which demonstrated that PDLIM2 suppression in PRAD cells induced upregulation of CDH1 mRNA expression and downregulation of VIM expression. These results indicate that PDLIM2 upregulation may fuel EMT and subsequent invasiveness, in PRAD cells. Among the six EMT signatures [26–31], we identified that Mathews et al.'s [28] signature was significantly associated with poor PFI in PRAD. These results indicate that PDLIM2 overexpression is highly associated with EMT signatures and worse prognosis.

Another significant aspect of this study is that PDLIM2 expression was found to be correlated with positive regulation of the immune response and negative regulation of the immune system process in PRAD. It is widely accepted that EMT is a critical mechanism by which tumor-associated immune cells promote tumor metastasis. Recently, growing evidence has demonstrated the involvement of PDLIM2 in immune responses. PDLIM2 acts as a tumor suppressor by inhibiting cancer-related genes and is involved in antigen presentation and T cell activation [55]. In mouse, inhibition of PDLIM2 resulted in increased lung cancer incidence and was reported to cause resistance against anticancer drugs and immunotherapeutic drugs, such as PD-1 blockers [34]. PDLIM2 suppresses Th1 and Th17 cell differentiation through inhibition of STAT3/4 and RelA in autoimmune disease [56], indicating that PDLIM2 plays a crucial role in T cell-mediated immune responses. Likewise, TME analysis demonstrated that the high-PDLIM2 expression group displayed a higher microenvironment score, immune score, and stromal score than the low-PDLIM2 expression group. In agreement with this, it has been reported that PRAD patients in the high immune infiltration group exhibit poorer survival [32]. This result may be the main reason for poor survival in patients in the high-PDLIM2 expression group.

It has historically baffled researchers to treat prostate cancer with immunooncology approaches due to its immunosuppressive microenvironment. To date, Sipuleucel-T, till now, is the only immunotherapeutic agent approved by the US Food and Drug Administration (FDA) for metastatic CRPC. However, no statistically significant difference in PFI was found between the treatment group and placebo group [57]. Therefore, new therapeutic strategies are needed. Meanwhile, it becomes necessary to find biomarkers to predict treatment effectiveness to ICIs in patients with PRAD.

In this study, we determined the correlation between PDLIM2 expression and TMB, MMR, and immune checkpoint genes. Theoretically, patients whose tumors have high TMB attain sensitivity to ICBs, contributing to a better outcome. Additionally, high expression of immune checkpoint genes is widely adopted as a predictor of the immunotherapeutic response rate. In this study, we demonstrated that the PDLIM2 abundant population had a higher TMB and exhibited higher expression of immune checkpoint genes. Consistent with our findings, a previous study [58] reported that TMB-low patients with PRAD had a better prognosis. McGrail et al. [59] reported that TMB failed to show predictive accuracy for ICB response in patients with PRAD. The discrepancy between our results and the literature could result from different variant calling algorithms and TMB calculation strategies [60]. A breakthrough study [61] demonstrated that the MMR-deficient tumors show a remarkable response to pembrolizumab, an immunotherapy targeting the PD-1 receptor. In this study, we found that PDLIM2 was negatively correlated with MSH2, PMS2, and EPCAM, indicating that PDLIM2 may play a role in PRAD by regulating MMR genes. Together, these findings suggest that PDLIM2 may be involved in PRAD progression and affect the efficacy of immunotherapy. Larger patient cohorts and subgroup analyses according to prostate cancer risk categories are needed to confirm our findings.

There were, regretfully but inevitably, several limitations to our study. First, we had to utilize data from TCGA and available public datasets to perform bioinformatic analysis due to an inadequate number of PRAD samples and the absence of RNA-seq data. Second, PDLIM2 abundance can induce EMT and suppress the immunogenicity of PRAD, indicating that these patients could potentially fail to benefit from immunotherapy and future studies are needed to validate our bioinformatic analyses. Third, in our study, the quantity of outcome events was limited on account of the relatively short follow-up time. Hence, the results should be considered preliminary, and longer-term studies are needed to identify more significant differences in the progression. Further studies are needed to confirm our hypothesis. Fourth, we used TMAs but not whole tissue sections, which may not reflect the full heterogeneity of primary PRAD. Fifth, we assessed PDLIM2 protein expression based on IHC, which is still a semiquantitative method. Thus, more quantitative examination is required.

## 5. Conclusions

In this study, our results suggested that PDLIM family genes might be significantly correlated with oncogenesis and the progression of PRAD. PDLIM2 may play an important role in EMT and cell infiltration, and thus, it is a potential prognostic biomarker for PRAD patients.

## Figures and Tables

**Table 1:** Association between low- or high-PDLIM2 mRNA expression and clinicopathological features in TCGA-PRAD cohort.

Characteristic	Low expression of PDLIM2	High expression of PDLIM2	*P*
*n*	249	250	
Age, *n* (%)			0.054^a^
≤60	123 (24.6%)	101 (20.2%)	
>60	126 (25.3%)	149 (29.9%)	
T stage, *n* (%)			0.150^a^
T2	105 (21.3%)	84 (17.1%)	
T3	136 (27.6%)	156 (31.7%)	
T4	5 (1%)	6 (1.2%)	
N stage, *n* (%)			0.057^a^
N0	180 (42.3%)	167 (39.2%)	
N1	31 (7.3%)	48 (11.3%)	
M stage, *n* (%)			1.000^b^
M0	226 (49.3%)	229 (50%)	
M1	1 (0.2%)	2 (0.4%)	
Primary therapy outcome, *n* (%)			<0.001^a^
PD	14 (3.2%)	14 (3.2%)	
SD	10 (2.3%)	19 (4.3%)	
PR	9 (2.1%)	31 (7.1%)	
CR	183 (41.8%)	158 (36.1%)	
Race, *n* (%)			0.215^a^
Asian	9 (1.9%)	3 (0.6%)	
Black or African American	28 (5.8%)	29 (6%)	
White	205 (42.4%)	210 (43.4%)	
Residual tumor, *n* (%)			0.051^b^
R0	169 (36.1%)	146 (31.2%)	
R1	62 (13.2%)	86 (18.4%)	
R2	3 (0.6%)	2 (0.4%)	
Zone of origin, *n* (%)			0.196^b^
Central zone	3 (1.1%)	1 (0.4%)	
Overlapping/multiple zones	53 (19.3%)	73 (26.5%)	
Peripheral zone	62 (22.5%)	75 (27.3%)	
Transition zone	6 (2.2%)	2 (0.7%)	
PSA (ng/ml), *n* (%)			0.801^a^
<4	212 (48%)	203 (45.9%)	
≥4	15 (3.4%)	12 (2.7%)	
Gleason score, *n* (%)			0.003^a^
6	28 (5.6%)	18 (3.6%)	
7	138 (27.7%)	109 (21.8%)	
8	31 (6.2%)	33 (6.6%)	
9	51 (10.2%)	87 (17.4%)	
10	1 (0.2%)	3 (0.6%)	

Abbreviations: CR: complete response; PD: progressive disease; SD: stable disease; PR: partial response; R0: no residual tumor; R1: microscopic residual tumor; R2: macroscopic residual tumor; PSA: prostate-specific antigen. All results are presented as *n* (%); ^a^chi-square test; ^b^Fisher test.

**Table 2:** Differential expression of PDLIM2 in cancerous and noncancerous prostate tissues.

	*n*	PDL1M2 expression		Chi-square value	*P* value
		High	Low		
Carcinoma	31	10	21	4.769	0.029^∗^
Normal	31	3	28		

^∗^Statistically significant (*P* < 0.05).

**Table 3:** Correlation between PDLIM2 protein expression and clinicopathological characteristics.

Variables	PDL1M2 expression		Total	*χ* ^2^	*P* value
	Low	High			
Age (year)				0.006	0.939
≤70	31	24	55		
>70	44	35	79		
Gleason score				7.047	0.029^∗^
6	21	7	28		
7	18	24	42		
>7	36	28	64		
T stage				4.009	0.045^∗^
T1, T2, T3	31	18	49		
T4	6	11	17		
N stage				4.063	0.044^∗^
N0	67	46	113		
N1	7	13	20		
34*β*E12				1.011	0.315
Low	70	52	122		
High	4	1	5		
AR				2.106	0.147
Low	3	0	3		
High	70	50	120		
CK5/6				1.511	0.219
Low	64	49	113		
High	2	0	2		
Ki67				4.514	0.034^∗^
Low	49	25	74		
High	23	26	49		
P504s				1.653	0.199
Low	5	1	6		
High	67	51	118		
P53				0.033	0.855
Low	52	36	88		
High	16	12	28		
P63				0.096	0.757
Low	71	52	123		
High	2	1	3		
PSA				0.926	0.336
Low	13	6	19		
High	60	46	106		
PSAP				1.653	0.199
Low	5	1	6		
High	67	51	118		
SMA				2.981	0.084
Low	54	40	94		
High	10	2	12		
TOPO-II				0.324	0.569
Low	12	11	23		
High	43	30	73		
ERG				0.159	0.690
Low	46	38	84		
High	11	11	22		
P170				0.067	0.795
Low	52	38	90		
High	5	3	8		

^∗^Statistically significant (*P* < 0.05). Abbreviations: AR: androgen receptor; PSA: prostate-specific antigen; PSAP: prostate-specific alkaline phosphatase; SMA: alpha-smooth muscle actin; TOPO-II: topoisomerase II; ERG: ETS-related gene.

## Data Availability

The datasets utilized and/or evaluated during this study are accessible upon reasonable request from the corresponding author.
